# Opacification of a scleral-sutured Akreos AO60 intraocular lens in the absence of concurrent or subsequent surgery: a case series

**DOI:** 10.1093/jscr/rjad181

**Published:** 2023-04-12

**Authors:** Harris Ahmed, Sam Subramanian, K V Chalam

**Affiliations:** Department of Ophthalmology, Loma Linda University Medical School, Loma Linda, CA, USA; Department of Ophthalmology, Loma Linda University Medical School, Loma Linda, CA, USA; Department of Ophthalmology, Loma Linda University Medical School, Loma Linda, CA, USA

## Abstract

Scleral-fixated intraocular lenses (SCIOLs) are an increasingly used option to place intraocular lenses in patients with compromised capsules. Akreos A060 is an acrylic hydrophilic lens that is commonly used for patients in need of SCIOL. As with other hydrophilic lenses, the Akreos A060 lens is associated with a risk of developing postoperative opacifications. To date, multiple case reports and case series have documented the development of opacifications in the Akreos A060 lens in the setting of subsequent intraocular surgery, most commonly after surgery involving gas or air, as commonly used in many routine retinal and corneal surgeries. Many theories have been proposed to explain this phenomenon, but none has been confirmed. This short case series presents two patients with Akreos A060 lenses who incidentally developed lens opacification in the absence of concurrent or subsequent intraocular surgery.

## INTRODUCTION

Many clinical situations may arise leading to compromised capsular bag support for a traditional intraocular lens such as trauma, pigmentary dispersion syndrome, pseudoexfoliation syndrome, conditions of hereditary ectopia lentis and prior complicated cataract surgery [1]. In such scenarios, one option among others is placing a scleral fixated lens (SCIOL). Compared to other options such as anterior chamber intraocular lenses (ACIOL) or iris-fixated intraocular lenses (IFIOL), SCIOLs are implanted in the sulcus and are less likely to be in contact with the iris and corneal endothelium [2]. Studies examining postoperative complications and visual acuity, however, have not shown the superiority of one technique over another [3–5]. Generally and overall, the literature confirms that SCIOLs are safe and effective, featuring low complication rates [6].

The CZ70BD (Alcon) and Akreos A060 (Bausch and Lomb) lenses are predominantly used for scleral fixation. These lenses feature suture eyelets that limit suture movement and IOL tilt and/or dislocation [1]. Compared to the CZ70BD lens, the Akreos A060 is hydrophilic and has four haptics with each haptic having its own eyelet [6]. The four-point fixation of the Akreos lens decreases the risk of IOL dislocation [6] compared to C270BD. The hydrophilicity of the Akreos A060 can be problematic, as it makes the lens susceptible to opacification, especially in the setting of intraocular gas or air fill that is used with many common retina and cornea surgeries [7]. Although rates of such opacification remain relatively low (with one study finding a rate of 2%), multiple cases of postoperative opacification of Akreos A060 lenses in the setting of retinal and corneal surgeries have been seen [7]. Such opacification is thought to occur due to the precipitation of calcium and phosphorous on the lens.

Many case reports and a case series have reported the presence of lens opacifications occurring in patients with hydrophilic lenses, in particular the Akreos A060 in patients who underwent additional ocular surgeries such as pars plana vitrectomy (PPV), Descemet stripping automated endothelial keratoplasty, Ahmed valve implantation and Ex-PRESS shunt implantation with transscleral drainage of a choroidal detachment [9–14]. Various theories have been proposed to explain why the phenomenon of lens opacification occurs in these patients. Theories include those related to air or gas causing localized damage to the IOL surface or increasing inflammation leading to opacification, UV blocking molecules and free radical formation from UV damage and vascular permeability in diabetics and subsequent expression of proteins that predispose to opacification; however, no theory has been confirmed [12, 15, 16].

In this short case series, we present the first cases of opacification in Akreos A060 lenses in the absence of concurrent or subsequent intraocular surgery.

## CASE SERIES

### Patient 1

On 11 November 2019, a 61-year-old male patient with a known history of proliferative diabetic retinopathy complicated by diabetic macular edema presented to the clinic. The patient had full pan-retinal photocoagulation and various intravitreal and sub-tenon treatments. He presented to the retina clinic with a chronic vitreous hemorrhage in the right eye with counting finger vision, necessitating a PPV with endolaser. The patient did well postoperatively, and by postoperative month one was 20/50, back to his baseline.

Seven months later, the patient underwent cataract surgery complicated by zonular instability and capsular bag rupture, ultimately leading to nuclear fragments falling posteriorly and vision of hand motion. A week later, a PPV was performed, the dislocated lens material was removed and the SCIOL was sutured uneventfully. During scleral fixated lens surgery, two 25-gauge sclerotomies were performed 2 mm posterior to the limbus at the 2, 4, 8 and 10 o’ clock meridians. The Gortex suture placed on the eyelets of the Akreos A060 lens was exteriorized uneventfully. A 23.5 D IOL was inserted through the superior corneal wound and placed in the ciliary sulcus. The Gortex sutures were exteriorized and tied. The IOL was secured in a central position, and the Gortex suture was buried in the sclera. Gas or air was not used during surgery.

**Figure 1:**
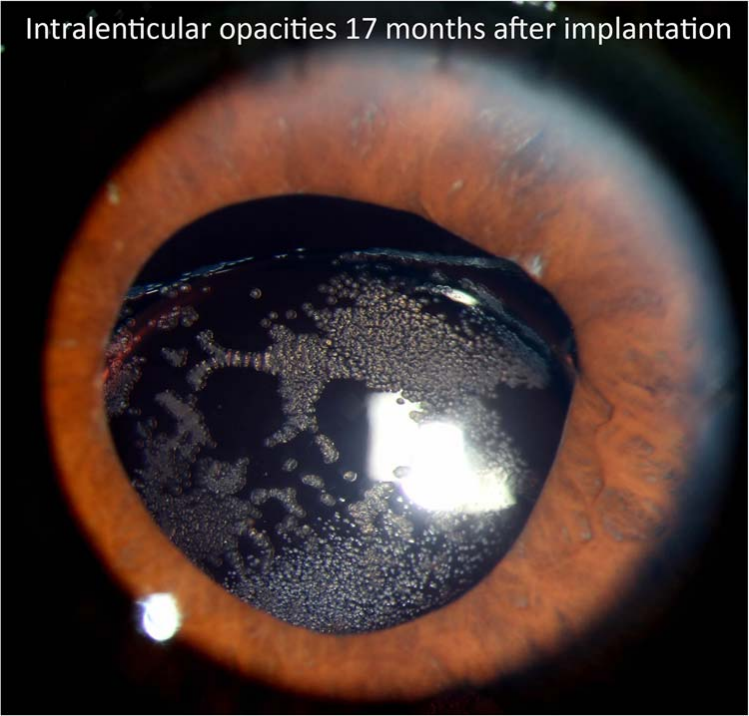
Slit lamp photograph illustrating lenticular opacities in patient 1.

**Figure 2:**
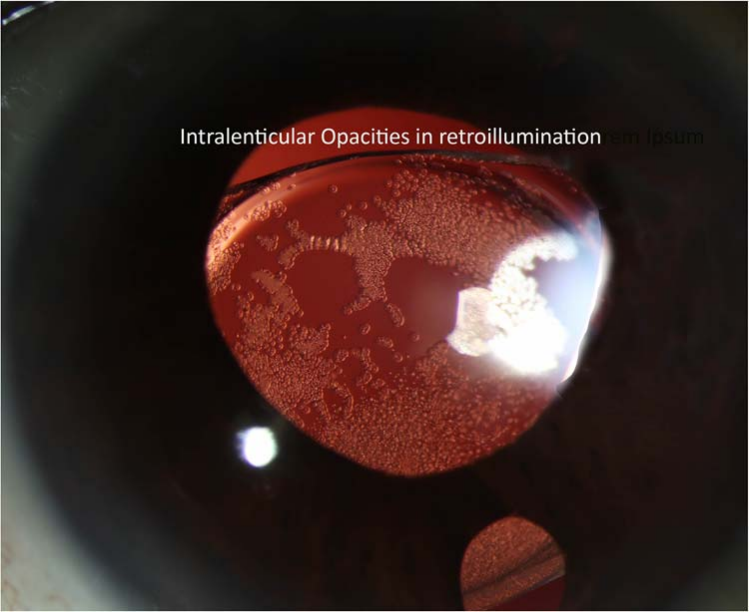
Slit lamp photograph highlighting lenticular opacities on retro-illumination.

The patient’s postoperative course was complicated by cystoid macular edema, which required a prolonged topical steroid taper and a single intravitreal preservative-free triesence injection. Ultimately, the patient returned to a baseline vision of ~20/50. The patient was routinely monitored for diabetic retinopathy, suspected glaucoma and an uneventful cataract surgery in the left eye. Seventeen months after SCIOL placement, the patient was noted to have multiple lenticular opacities on slit-lamp examination with both direct and retro illumination (Figs 1 and 2). However, the visual acuity remained stable at 20/50.

### Patient 2

**Figure 3:**
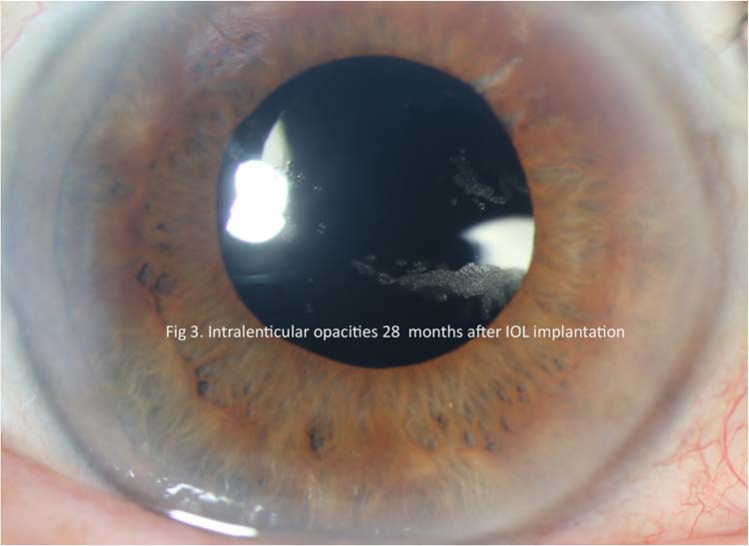
Slit lamp photograph illustrating lenticular opacities in patient 2.

**Figure 4:**
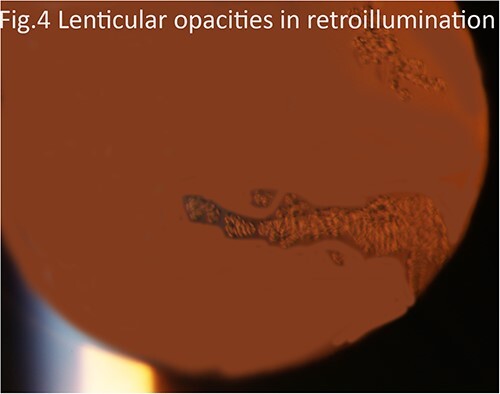
Slit lamp photograph highlighting lenticular opacities on retro-illumination.

A 72-year-old female with a history of pseudoexfoliation glaucoma in both eyes and pseudophakia of the left eye (performed at an outside center) presented to our clinic with blurred vision and visual distortion in the left eye. On examination, the BCVA was HM with inferior dislocation of a previously placed IOL. Dislocated IOL was removed, and the new SCIOL was sutured in a fashion similar to that described above. The surgery was completed without complications or the use of air or gas. The patient's visual acuity improved to 20/60 by postoperative week 1; however, the course was complicated by cystoid macular edema three months after surgery. The patient received three preservative-free intravitreal triescence injections to control the edema over a span of 9 months. The CME resolved completely, and BCVA improved to 20/25. The patient struggled with glaucoma drop compliance and experienced further thinning of the retinal nerve fiber layer and poor intraocular pressure control. Twenty-four months after the initial surgery, the lens opacities traversed the visual axis (Figs 3 and 4). Visual acuity remained stable despite opacities, and the decision to observe opacifications was made.

## DISCUSSION

Opacification of hydrophilic lenses, and in particular the Akreos A060 lens, has been well documented in the literature. Given the rise in vitrectomies being performed in the elderly population, this complication and the possible situations it may arise in are important for retina surgeons to be aware of [17].

Belin *et al*. reviewed 262 eyes of 257 patients who received Akreos A060 lenses and found that five patients (2% rate) developed opacifications [8]. All five were exposed to gas or air, four underwent concurrent or subsequent DSAEK and one underwent multiple vitrectomies with SF6 gas. Of these five, three required lens exchange, while the other two were observed. As such, Belin *et al*. recommended caution when using the Akreos A060 lens in patients who may require PPV or DSAEK in the future. Among our patient population, such opacifications have been noted in two of 126 patients (1.6%), a rate comparable to that reported by Belin *et al*. Notably, one patient in the Belin series featured multiple intravitreal injections for macular edema, similar to our patients; however, their patient also underwent simultaneous DSAEK involving an air injection along with Akreos A060 lens implantation.

Cao *et al*. presented a case of a patient with severe nonproliferative diabetic retinopathy who received an Akreos AO acrylic hydrophilic lens in both eyes and subsequently required vitrectomy in one eye, ultimately developing visually significant opacification in the eye that underwent vitrectomy [10]. Additionally, the authors found that four out of six patients with opacifications following the use of an Akreos AO lens had diabetes. As such, the authors posit that diabetes and inflammatory states, such as after vitrectomy, can serve as risk factors for the development of opacification in patients with Akreos hydrophilic lenses. Although Cao *et al*. stated that caution should be used with hydrophilic acrylic lenses in patients with diabetes, they admitted that a correlation between diabetes and the development of opacification could not be established. The impact of diabetes was equivocal in our patients, as only one of the two patients had diabetes. In the five reported cases presented by Belin *et al*., none had a history of diabetes or intraocular inflammation.

Our cases revealed that the mechanisms behind opacification are not well understood and represent an interplay of many factors beyond just air or gas trauma and exposure, vasculopathic risk factors or inflammatory histories. Additionally, our case highlights that there may be multiple independent or synergistic pathways that can lead to opacification. Only one of our patients had diabetes, none had a known inflammatory history and none underwent concurrent or subsequent intraocular surgeries with gas or air exposure after the placement of their Akreos A060 lenses yet both developed opacifications. Interestingly, however, both patients' postoperative courses were complicated by cystoid macular edema, suggesting that our patients had a predisposition to enter an inflammatory state. The role of inflammation and the inflammatory state contributing to a higher risk of developing opacification is disputed. Additionally, our patients both received intravitreal triesence injections that were another variable introduced; however, the formulation of these injections was preservative free, making it unlikely to have contributed to the formation of the intraocular opacities.

Gregori *et al*. presented a unique case of a patient with an existing Akreos A060 lens who underwent retinal detachment repair and silicone oil placement and subsequently developed opacification [18]. Their case demonstrates that opacifications may occur in other scenarios beyond exposure to gas or air. In our study, the patients received intravitreal triamcinolone injections. It is possible that some properties or elements of the process of intravitreal injection, such as secondary inflammation or a property inherent to triamcinolone, may similarly cause disruption to the intraocular lenses, leading to a higher risk of opacification.

Our cases add to the literature in that patients need not be exposed to gas or air in concurrent or subsequent surgery to develop opacification. Such opacifications may occur in a small subset of patients for reasons that are presently unclear and require further investigation. The roles of vasculopathic or inflammatory history in the risk of developing opacification remain unclear. Additionally, the presence of opacifications may not always be visually significant or necessitate lens exchange, as demonstrated in our cases and others in the literature, such as those presented by Belin *et al*.

In conclusion, we described the first series of cases of opacification in Akreos A060 lenses in the absence of concurrent or subsequent intraocular surgery.

## Data Availability

The data that support the findings of this study are available on request from the corresponding author, KVC. The data are not publicly available due to containing information that could compromise the privacy of research participants.
